# Wild-type C-Raf gene dosage and dimerization drive prostate cancer metastasis

**DOI:** 10.1016/j.isci.2023.108480

**Published:** 2023-11-17

**Authors:** Lisa Ta, Brandon L. Tsai, Weixian Deng, Jihui Sha, Grigor Varuzhanyan, Wendy Tran, James A. Wohlschlegel, Janai R. Carr-Ascher, Owen N. Witte

**Affiliations:** 1 Department of Molecular and Medical Pharmacology, University of California, Los Angeles; Los Angeles, CA 90095, USA; 2 Department of Human Genetics, University of California, Los Angeles; Los Angeles, CA 90095, USA; 3 Department of Biological Chemistry, David Geffen School of Medicine, University of California, Los Angeles; Los Angeles, CA 90095, USA; 4 Department of Microbiology, Immunology, and Molecular Genetics, University of California, Los Angeles; Los Angeles, CA 90095, USA; 5 Department of Internal Medicine, Division of Hematology/Oncology, University of California, Davis, Sacramento, CA 95817, USA; 6 Department of Orthopedic Surgery, University of California, Davis; Sacramento, CA 95817, USA; 7 Molecular Biology Institute, University of California, Los Angeles; Los Angeles, CA 90095, USA; 8 Eli and Edythe Broad Center of Regenerative Medicine and Stem Cell Research, University of California, Los Angeles, Los Angeles, CA 90095, USA; 9 Jonsson Comprehensive Cancer Center, University of California, Los Angeles; Los Angeles, CA 90095, USA; 10 Parker Institute for Cancer Immunotherapy, University of California, Los Angeles; Los Angeles, CA 90095, USA

**Keywords:** Biochemistry, Molecular biology, Cancer, Proteomics, Transcriptomics

## Abstract

Mutated Ras and Raf kinases are well-known to promote cancer metastasis via flux through the Ras/Raf/MEK/ERK (mitogen-activated protein kinase [MAPK]) pathway. A role for non-mutated Raf in metastasis is also emerging, but the key mechanisms remain unclear. Elevated expression of any of the three wild-type Raf family members (C, A, or B) can drive metastasis. We utilized an *in vivo* model to show that wild-type C-Raf overexpression can promote metastasis of immortalized prostate cells in a gene dosage-dependent manner. Analysis of the transcriptomic and phosphoproteomic landscape indicated that C-Raf-driven metastasis is accompanied by upregulated MAPK signaling. Use of C-Raf mutants demonstrated that the dimerization domain, but not its kinase activity, is essential for metastasis. Endogenous Raf monomer knockouts revealed that C-Raf’s ability to form dimers with endogenous Raf molecules is important for promoting metastasis. These data identify wild-type C-Raf heterodimer signaling as a potential target for treating metastatic disease.

## Highlights

• Elevated C-Raf promotes metastasis in a gene dosage-dependent manner
• Ectopic C-Raf’s kinase activity is dispensable in the presence of endogenous Raf
• C-Raf forms dimers with endogenous Raf molecules to promote metastasis

## Graphical abstract

**Figure:**
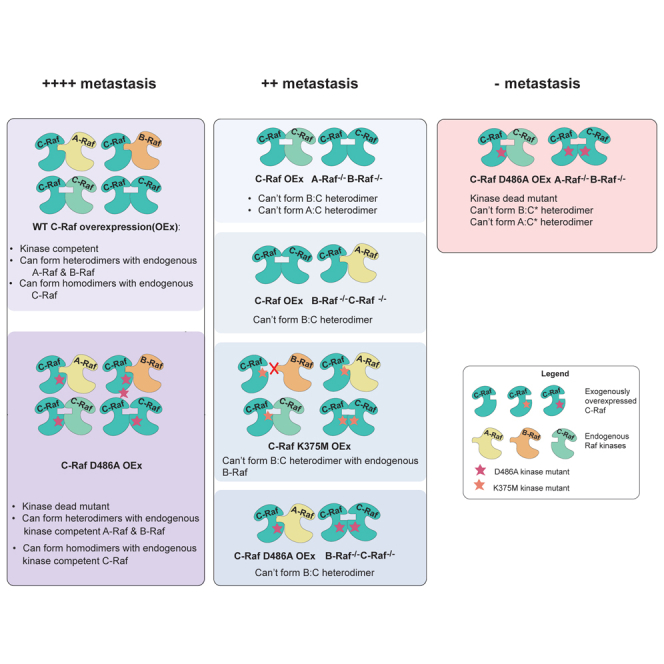

## Introduction

Metastasis is the primary cause of cancer-related deaths and remains a significant clinical challenge.^1^ Tumor metastasis involves a multi-step cascade that requires tumor cells to survive a variety of environmental stressors. This process includes tumor cell invasion of the basement membrane, intravasation into the blood stream or lymphatic vessel, survival in the circulation, extravasation, and colonization at a secondary site.^2^ The Ras/Raf/MEK/ERK (mitogen-activated protein kinase [MAPK]) cascade is a well-studied mediator of metastasis. Ras and B-Raf are commonly mutated upstream regulators of MAPK signaling in cancer cells.^3^^,^^4^ Targeting activating mutations like BRAFV600E with small-molecule inhibitors has been successful and frames kinases as viable targets for therapeutic development.^5^^,^^6^^,^^7^

Though much focus has been on mutated Raf, we and others have shown that wild-type (WT) Raf plays an important role in promoting cancer and metastasis.^8^^,^^9^^,^^10^^,^^11^ Using a functional screen, we previously found that all three WT Raf kinases (C-, A-, and B-Raf) can promote metastasis of immortalized prostate cells in an *in vivo* metastatic model.^11^ In concert with the screen, interrogation of human tissue microarray samples demonstrated enrichment of Raf kinase proteins in metastatic samples compared to localized tumors and normal tissue.^11^ Among the three kinases, C-Raf drove the most penetrant metastatic phenotype. Whether C-Raf drives metastasis via its gatekeeping role in the MAPK pathway or via other interactions remained unknown.

Raf kinase activation is a complex process that requires a series of dephosphorylation and phosphorylation events that prime Raf molecules for dimerization with other Raf monomers^12^ (review). This homo- or heterodimerization with other Raf kinase-competent monomers is critical for propagating MAPK signaling. Specific dimer combinations can determine the aggressiveness of cancer phenotypes. Recent work pointed to WT A-Raf:C-Raf heterodimers as key regulators in K-Ras-driven tumor growth.^13^ Venkatanarayan and colleagues found that K-Ras mutant cells contained more A-Raf:C-Raf than B-Raf:C-Raf dimers. Further work done by this group showed that C-Raf’s dimerization function, but not its kinase activity, is important in driving malignant phenotypes in an *in vitro* setting. Their study extensively explored C-Raf dimerization in the context of Ras-driven disease. However, the relevance of WT C-Raf’s role has not been explored in a non-mutant Ras context. Additionally, many of the key findings were modeled in a soft agar assay, necessitating further testing of C-Raf’s dimerization and kinase functions in promoting metastasis in an *in vivo* setting.

We performed a systematic investigation of WT C-Raf’s functions in driving cancer metastasis in a WT K-Ras context. We chose to interrogate C-Raf’s function as an archetype of the Raf kinases due to its more potent metastatic phenotype in our *in vivo* models.^11^ To model metastasis, an intracardiac injection mouse model was used, which captures multiple steps of the metastatic cascade, including resistance to anoikis and cell death, seeding ability in distant tissues, and most importantly bypasses sequestration of disseminated cells in the lungs^14^ (review). Utilizing orthogonal mass spectrometry, transcriptomic analyses, various mutants targeting specific Raf functions, and select Raf monomer genetic deletions, we show that even subtle increases in C-Raf protein expression can drive metastasis. Exogenously added C-Raf-driven metastasis is dependent on its dimerization domain. Overexpressed C-Raf dimerizes with endogenous A-Raf and B-Raf to drive metastasis. This work highlights the importance of C-Raf heterodimerization and gene dosage in driving metastatic cancer and points to the potential of perturbing these interactions for better therapeutic outcomes.

## Results

### C-Raf overexpression enables transformation and metastasis of two immortalized prostate cell lines

**Figure 1:**
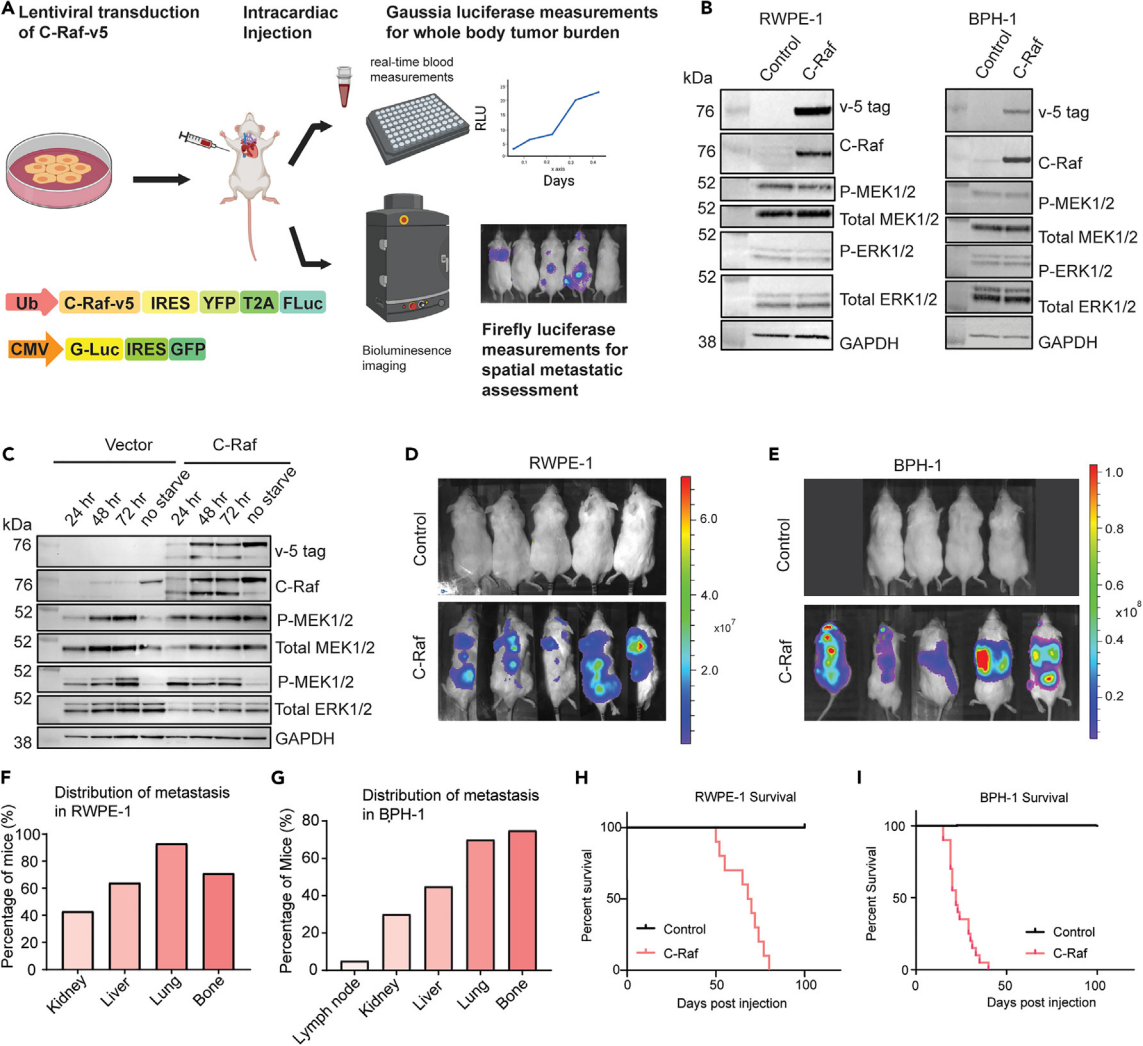
C-Raf overexpression drives metastasis of two immortalized prostate cell lines (A) Schematic of model system and workflow used to study C-Raf overexpression *in vivo*. RAF1 and reporter genes were expressed in immortalized prostate lines and injected into mice via intracardiac injection followed by serial blood measurements and bioluminescence imaging (BLI). Firefly luciferase reporter system was used to assess spatial metastatic activity via BLI. Gaussia luciferase reporter system was used to assess whole-body tumor burden measurement via real-time blood measurements. (B) Western blot analysis demonstrating C-Raf-v5 overexpression and subsequent downstream effectors including P-MEK1/2 and P-ERK1/2 in RWPE-1 and BPH-1 cell lines. (C) Western blot analysis of time point serum starvation of BPH-1 cells (D) BLI imaging at 28 days post RWPE-1 injection. (E) BLI imaging at 14 days post-injection of BPH-1. (F) Distribution of macroscopic tumors across 12 mice with C-Raf overexpression and vector control in RWPE-1 cells. (G) Distribution of macroscopic tumors across 30 mice with C-Raf overexpression and vector control in BPH-1 cells. (H) Kaplan-Meier curve of mice harboring RWPE-1 vector control vs. C-Raf overexpression cells (n = 10/group, log rank (Mantel Cox) test p = 0.0001). (I) Kaplan-Meier curve of mice harboring BPH-1 vector control vs. C-Raf-overexpression cells (n = 10/group, log rank (Mantel Cox) test, p = 0.0001).

To better understand the mechanism by which non-mutated C-Raf promotes metastasis, we tested C-Raf heightened expression in two immortalized prostate epithelial cell lines, benign prostatic hyperplasia (BPH)-1, and RWPE-1. These lines are immortalized by SV40 and HPV18, respectively. Neither line forms metastasis *in vivo*. C-Raf-overexpressing human cells were administered to immune-defective non-obese diabetic (NOD) scid gamma (NSG) mice via intracardiac injection to monitor metastatic dissemination (Figure 1A). Both cell lines were engineered to express firefly and Gaussia luciferase to track the spatial distribution of metastatic tumors^15^ and to quantify whole-body tumor burden, respectively (Figure 1A).^16^ Static measurements of C-Raf overexpression in serum conditions did not result in significant changes in MEK1/2 and ERK1/2 phosphorylation levels (Figure 1B). This observation corroborates other studies that interrogated WT B-Raf and A-Raf overexpression.^17^^,^^18^ When we interrogate BPH-1 vector and C-Raf overexpression cells under serum starvation conditions, MAPK downstream activation appeared activated as measured by P-MEK1/2 and P-ERK1/2 following longer periods of serum starvation (Figure 1C). This may be due to release of feedback inhibition compared to no-starvation control. The presence of C-Raf overexpression also consistently promoted downstream MAPK as measured by P-MEK1/2 and P-ERK1/2 compared to no-starvation control and appears slightly enhanced at 24 h compared to vector control. Naive RWPE-1 and BPH-1 cells did not form tumors upon intracardiac injection for the duration of the study (100 days). In contrast, C-Raf overexpression caused both cell lines to develop robust metastasis in all injected mice (Figures 1D and 1E). C-Raf drove metastasis to multiple visceral organs and bone in both RWPE-1 and BPH-1 models (Figures 1F and 1G). The most common site of metastasis in prostate cancer patients is bone.^19^ Bone was consistently one of the most common metastatic sites in our models, with greater than 60% of animals harboring metastatic tumors in this location in RWPE-1 and greater than 70% in BPH-1 model (Figures 1F and 1G). Whereas control mice survived for the duration of the study (100 days), mice harboring RWPE-1 C-Raf cells became moribund on average 42 days post-injection (Figure 1H) and mice with BPH-1 C-Raf died on average 30 days post-injection (Figure 1I). To distinguish C-Raf’s tumor initiation from its metastatic potential, we conducted a subcutaneous tumor initiation experiment in mice. After 18 days, subcutaneous tumors were surgically resected, and animals were monitored for metastatic sites beyond the injection site using bioluminescence imaging (BLI) after 4 weeks (Figure S1). Interestingly, vector control group did not develop any tumors for the duration of the study. Notably, C-Raf facilitated metastasis to the lungs and bone in mice, mirroring locations seen in prostate cancer metastasis. In essence, heightened C-Raf expression consistently induces metastasis in mice, resembling typical prostate cancer metastasis locations.

### RAF1 drives metastasis and mortality in a gene dosage-dependent manner

**Figure 2:**
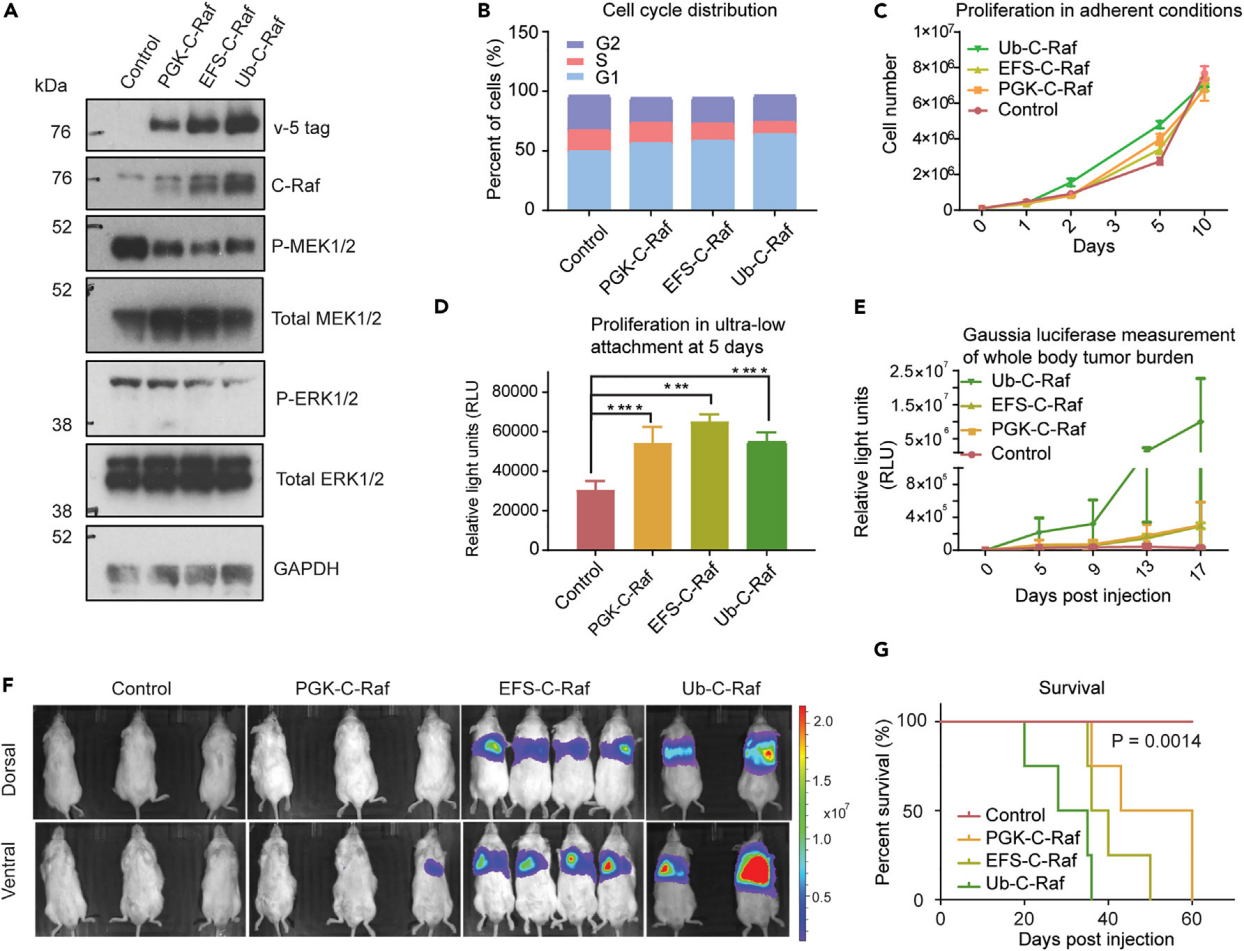
RAF1 drives metastasis and mortality in a gene dosage-dependent manner (A) Western blot depicting increasing C-Raf protein expression driven by promoters of varying strength in BPH-1 cells. (B) Cell cycle analysis using propodium iodide across cell lines with increasing C-Raf expression in adherent culture conditions. Vector (G1: 49.7%, S: 17.5%, G2: 29%) PGK (G2: 56.4%, S: 17.0%, G2: 20.9) EFS (G1: 58.5%, S: 14.4%, G2: 21.5%) Ub (G1: 64%, S: 10.3%, G2: 22.1%). (C) Proliferation as measured by trypan blue staining over 10 days in adherent conditions. Data are represented as mean ± SEM. (D) Proliferation of cells measured by cell titer glo assay of C-Raf increasing expression cell lines in anchorage-independent conditions at 5 days (unpaired t test, p = 0.0002, 0.001, 0.0001 from left to right). (E) Gaussia luciferase blood measurements as a surrogate marker of tumor burden over 17 days. Data are represented as mean ± SEM. (F) BLI imaging of C-Raf dosage lines at 28 days post-intracardiac injection. (G) Kaplan-Meier curve of mice harboring cells with increasing C-Raf expression (n = 4/group, Log rank (Mantel-Cox) test p = 0.0014, all groups significantly different) Data are represented as mean ± SEM.

C-Raf protein can accumulate above normal levels via genetic amplification or dysregulation at the mRNA and protein levels.^4^^,^^6^^,^^20^ We chose to proceed with the BPH-1 cell line for our mechanistic studies due to the expedited *in vivo* growth kinetics. To determine the threshold of C-Raf protein necessary to drive metastasis, C-Raf gene dosage was exogenously manipulated via various strength promoters in BPH-1 cells. C-Raf protein was expressed at approximate levels of ∼2-fold, 3-fold, and 4-fold above endogenous levels driven by EF-1α short (EFS), Pgk-1 (PGK), and Ubiquitin (Ub) promoters, respectively (Figure 2A) (quantification in Figure S2A) using an empty Ub promoter as control. Increasing C-Raf protein expression did not increase downstream phospho-MEK1/2 and phospho-ERK1/2 levels as well as their corresponding total MEK1/2 and ERK1/2 (Figure 2A). This may be because MEK1/2 and ERK1/2 phosphorylation levels are likely constrained by negative feedback regulation.^21^

We performed cell cycle analysis by flow cytometry to check whether C-Raf overexpression enhanced cell proliferation to promote metastasis. C-Raf promoted accumulation of cells in the G1 phase of the cell cycle in a gene dosage-dependent manner under adherent conditions. Although dose-dependent increase of C-Raf did not result in significant growth differences under adherent conditions *in vitro* (Figures 2B and 2C), it significantly enhanced proliferation in an anchorage-independent environment (Figure 2D). To verify this dose-dependent effect *in vivo*, we injected mice and quantified metastatic tumor burden using Gaussian blood measurements and BLI (Figures 2E and 2F). Increasing dosage of C-Raf resulted in a dose-dependent increase in metastatic burden (Figures 2E and 2F) and a concomitant decrease in survival (Figure 2G). Only 2-fold higher expression above endogenous was sufficient to drive metastasis. Further increase in C-Raf levels conferred an additional advantage to cells and promoted more aggressive metastasis. These results indicate that even modest increases in C-Raf protein expression can promote malignancy and metastasis.

### C-Raf overexpression increases MAPK pathway flux and is associated with metastasis

C-Raf is a serine/threonine protein kinase and canonically functions via altering the phosphorylation status of downstream substrates and their physical association with other proteins^22^ (review). Heightened C-Raf expression might amplify other functions beyond the MAPK pathway. To explore the activity and downstream effects of C-Raf overexpression, we analyzed the phosphoproteome of parental C-Raf-overexpressing cells compared to vector control using tandem mass tag (TMT) isobaric labeling and phosphoproteomic analysis.^23^ TMT labeling coupled with phosphorylation enrichment and mass spectrometry allows quantification of the relative abundance of phosphorylated peptides^24^ (Figure S1A). C-Raf induced drastic changes to the phosphoproteome as expected. The PC1 spread in principal-component analysis (PCA) captured 57.7% of the variance that primarily separates C-Raf and vector groups (Figure S1B). 48,029 distinct phospho-peptides were detected in C-Raf cells compared to control when adjusted to their corresponding protein abundance, indicating a robust restructuring of the phosphoproteome.

We performed inferred kinase activity (IKA) analysis^25^ to identify kinases that have upregulated activity based on the quantification of known phosphorylated substrates (Figure S3C). IKA analysis showed significant upregulation of C-Raf, CDK1, and CDK2, which are involved in cell division and proliferation. CDK2 has been shown to be regulated by MAPK activation^26^^,^^27^^,^^28^ (Figure S3D). VRK1 and VRK2, two serine/threonine kinases in the same family that regulate chromatin remodeling and cell cycle progression, are also significantly enriched.^29^ Though neither kinase has been shown to have a strong relationship with C-Raf signaling, increased mTOR signaling via MAPK and PI3K pathway crosstalk has been shown to regulate VRK1 and VRK2 activity.^30^ IKA analysis also provided information on overall pathway/complex upregulation. C-Raf samples demonstrated significant upregulation of Ras/Raf signaling compared to vector control (Figure S3E). Other statistically significant pathways include several chromatin and histone-modifying complexes, the DNA damage binding complex, and the ubiquitin E3 ligase complex (Figure S3E). Increased flux through chromatin remodeling pathways coincides with increased VRK1 and VRK2 activity. Taken together, these data highlight that overexpression of C-Raf increases MAPK flux and possible downstream crosstalk with PI3K pathway effectors.

### Transcriptional targets of the MAPK pathway are upregulated in metastatic C-Raf-driven tumors

C-Raf signals through MEK and ERK to drive widespread transcriptional change^22^^,^^31^ (review). To obtain an unbiased evaluation of the effects of ectopic C-Raf expression on the transcriptome, mRNA sequencing was performed in vector control, parental C-Raf-overexpressing cells, and C-Raf metastasis-derived cell lines (Figure S4A). Macroscopic tumors were resected from the bone, lymph node, spine, and thymus and cultured over a period of a week to establish metastasis-derived lines. Differential expression analysis revealed that C-Raf drives a distinct transcriptional program, with over 650 differentially abundant genes compared to parental cells (Figure S4B). Comparisons performed between pooled metastasis-derived cell lines and their C-Raf-overexpressing parental lines identified over 600 differentially regulated genes. Combined analysis of vector control, parental C-Raf, and C-Raf metastasis-derived lines demonstrated that 46 genes were perturbed in the same direction (increasing or decreasing) in a stepwise fashion from vector control to parental C-Raf, and to C-Raf metastasis-derived lines (Figure S4C). Out of the 18 genes that demonstrated such stepwise increase in expression, 9 were known targets of Ras/Raf signaling (Figure S4C). In concert with the phosphoproteomic data, transcriptional analysis of C-Raf overexpression demonstrated increased MAPK pathway activation.

Each cell line was scored for key cancer hallmark signature gene sets to evaluate which biological processes are changed in C-Raf metastatic lines compared to the C-Raf parental and vector control lines.^32^^,^^33^ K-Ras signaling was upregulated in both the C-Raf parental and metastatic lines as compared to the vector control (Figure S2D). Transforming growth factor β (TGF-β) signaling, a known downstream target of MAPK signaling,^34^ was also upregulated in both C-Raf parental and C-Raf-derived metastasis lines (Figure S4D). Notch signaling is significantly suppressed with C-Raf overexpression in C-Raf parental and metastasis-derived lines.^35^ There was no clear evidence of MAPK-independent C-Raf processes, like transcriptomic upregulation of anti-apoptotic pathways with MST2 or ASK1. Overexpression of C-Raf predominantly drives an MAPK transcriptional program associated with tumor metastasis.

### Mutation in the dimerization domain ablates C-Raf’s metastatic-promoting effects

**Figure 3:**
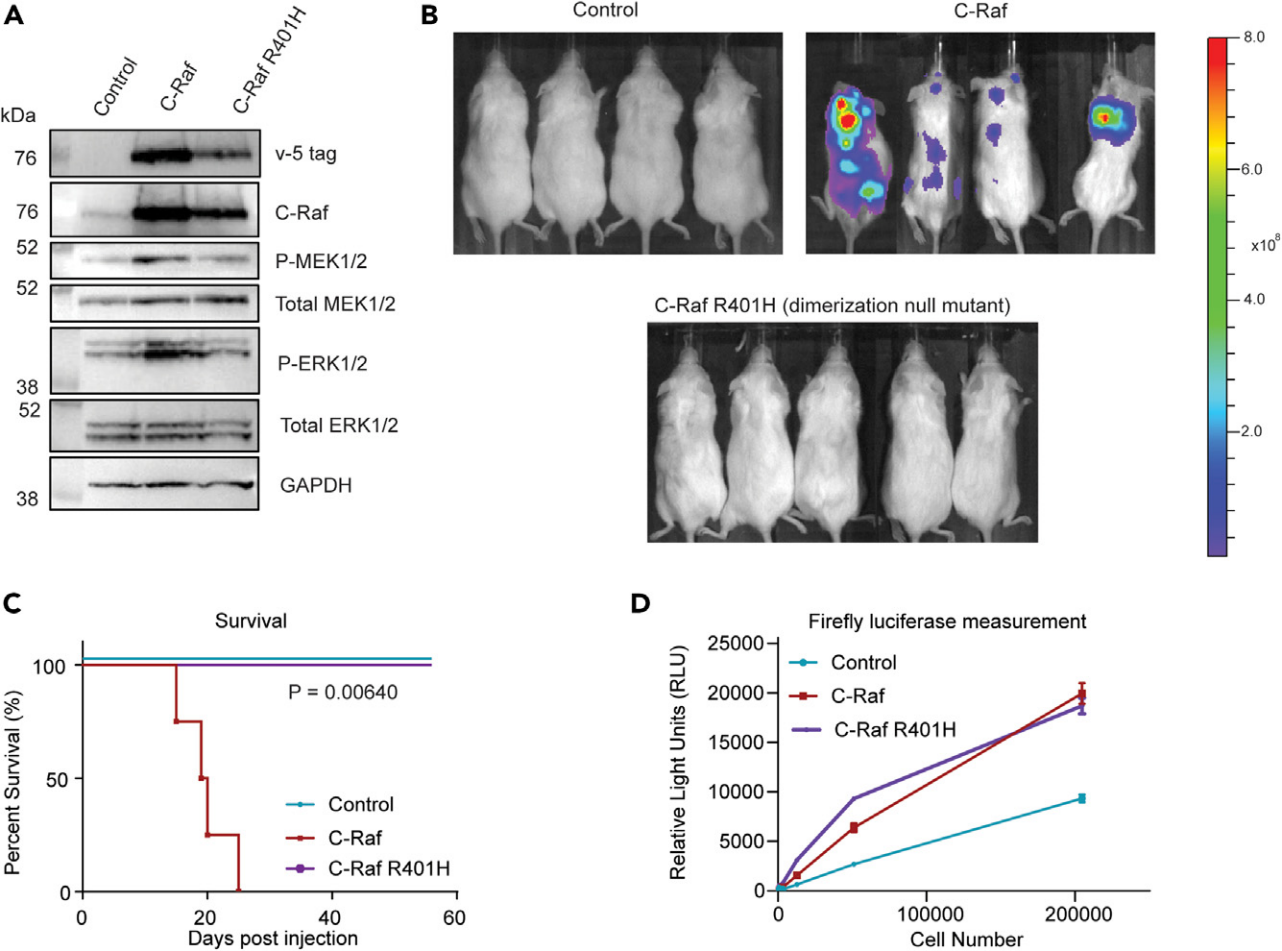
Mutation of C-Raf dimerization domain ablates metastatic ability (A) Western blot analysis of C-Raf dimerization mutant overexpression compared to WT C-Raf in BPH-1 cells. (B) BLI 28 days post-injection of WT C-Raf compared to C-Raf dimerization null (R401H) and vector control. (C) Kaplan-Meier curve of mice harboring WT C-Raf and mutant cells (n = 5/group, log rank (Mantel-Cox) test p = 0.0064, all groups significantly different). (D) *In vitro* cell titration detected using D-luciferin substrate to confirm firefly surrogate tumor measurements *in vivo.* Data are represented as mean ± SEM.

Dimerization is a crucial step in C-Raf’s activation sequence and is necessary for WT Ras-dependent Raf kinase activation as reviewed in Lavoie et al. 2015.^12^ To test whether dimerization is necessary for C-Raf-induced metastasis, a dimerization-null mutant, R401H, was introduced into BPH-1 cells. R401H mutation is in the RKTR motif within the αC-helix region of the dimerization interface.^36^ Mutation of arginine to histidine has been demonstrated to significantly diminish kinase activity and inhibit dimerization with other Raf monomers.^37^^,^^38^^,^^39^ R401H expression resulted in a modest decrease in phospho-MEK1/2 and phospho-ERK1/2 (Figure 3A). Importantly, cells expressing the C-Raf R401H mutant did not produce metastasis *in vivo* (Figure 3B). Mice harboring C-Raf R401H cells did not exhibit any metastatic burden at 10 weeks post-injection (Figure 3C). Cell titration was performed *in vitro* using D-luciferin substrate to ensure that the cells had adequate reporter gene output *in vivo*. All cells were positive for expression of firefly luciferase (Figure 3D). These results indicate that protein-protein interactions at the dimerization interface are required for C-Raf’s ability to drive metastasis.

### Combination knockout of Raf paralogs diminishes C-Raf-driven metastasis

**Figure 4:**
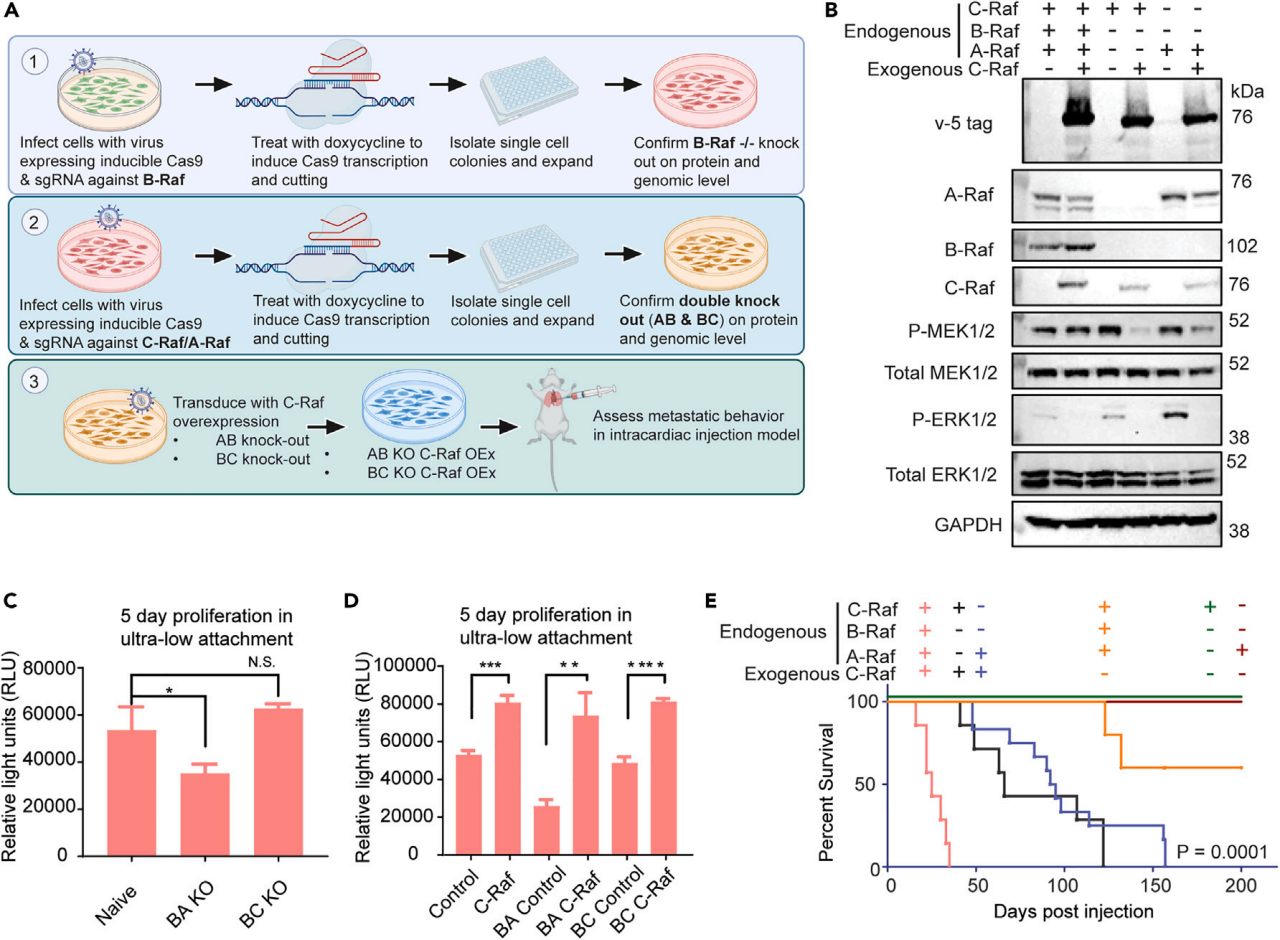
Combination knockout of Raf family members diminishes C-Raf-driven metastasis (A) Schematic of double knockout cell line generation starting from viral transduction, inducible Cas9 induction via doxycycline at 1 μg/mL, single-cell cloning, and subsequent confirmation and sequential generation of double knockout line. (B) Protein level confirmation of endogenous KO combinations A-Raf and B-Raf (AB) or B-Raf and C-Raf (BC) and their corresponding C-Raf-overexpression lines. (C) Proliferation of double knockout cell lines compared to parental control in anchorage-independent conditions (n = 3/group, unpaired t test, p = 0.04). (D) Proliferation in anchorage-independent conditions with C-Raf overexpression (unpaired t test from left to right, p = 0.0004, 0.0027, 0.0001). (E) Combined survival curve of mice harboring cells with C-Raf overexpression in BPH-1 cells and BPH01 AB & BC KO cell lines (n = 8/group log rank (Mantel-Cox) test, p = 0.0001, all groups significantly different). Data are represented as mean ± SEM.

To determine which dimer pairs are necessary and sufficient to contribute to C-Raf-driven metastasis, we knocked out multiple combinations of endogenous Raf kinases. We found that triple knockout of A-, B-, and C-Raf was lethal to BPH-1 cells. As a follow-up strategy, two combinations of knockouts were selected based on prior literature highlighting the importance of B-Raf:C-Raf and A-Raf:C-Raf heterodimers. B-Raf and C-Raf have been shown to be the preferential dimer pair, while A-Raf and C-Raf dimerization has been shown to mediate K-Ras-driven malignancy.^13^^,^^40^^,^^41^ Two combinations with knockout of B-Raf were made. A clonal B-Raf-knockout line was first generated using CRISPR/Cas9^42^ and sequentially modified by either A-Raf or C-Raf knockout (Figures 4A and 4B). The resulting endogenous A-Raf^−/−^; B-Raf^−/−^ and B-Raf^−/−^; C-Raf^−/−^ cell lines are subsequently referred to as AB KO and BC KO, respectively. Exogenous C-Raf overexpression in AB KO and BC KO cell lines decreased phospho-MEK1/2 and phospho-ERK1/2 levels (Figure 4B), indicating robust negative feedback relative to vector control. While AB KO cells proliferated at a slower rate than naive BPH-1 cells, BC KO showed no growth difference in *in vitro* anchorage-independent conditions (Figure 4C). Consistent with our previous results (Figure 2D), C-Raf overexpression significantly increased proliferation in ultra-low attachment conditions, which was not reduced by BA or BC knockout (Figure 4D).

Although BA and BC KO did not reduce C-Raf-induced heightened proliferation *in vitro*, mice administered with either C-Raf AB KO or C-Raf BC KO cells exhibited drastically reduced metastatic burden compared to C-Raf overexpression only, as reflected in their survival (Figure 4E). AB KO in C-Raf-overexpressing cells significantly extended survival by ∼50 days compared to the C-Raf-overexpression-only group. BC KO in C-Raf-overexpressing cells also extended survival compared to C-Raf-overexpression-only group by ∼110 days (Figure 4E). Based on the combinatorial KOs, in C-Raf BC KO cells, the only available monomers to form dimers are endogenous A-Raf and exogenous C-Raf. In the C-Raf AB KO cells, the only available monomers are endogenous and exogenous C-Raf. The aforementioned metastatic phenotypes suggest the ability to form either A-Raf:C-Raf heterodimers and/or C-Raf homodimers is sufficient to drive metastasis albeit with longer latency. The ablation of B-Raf in both KO groups severely dampened C-Raf-driven metastasis. Therefore, these data provide some support that B-Raf:C-Raf dimers may promote aggressive metastatic disease in our model.

### Mutation of the DFG motif and the ATP binding site in C-Raf’s kinase domain result in different metastatic phenotypes

**Figure 5:**
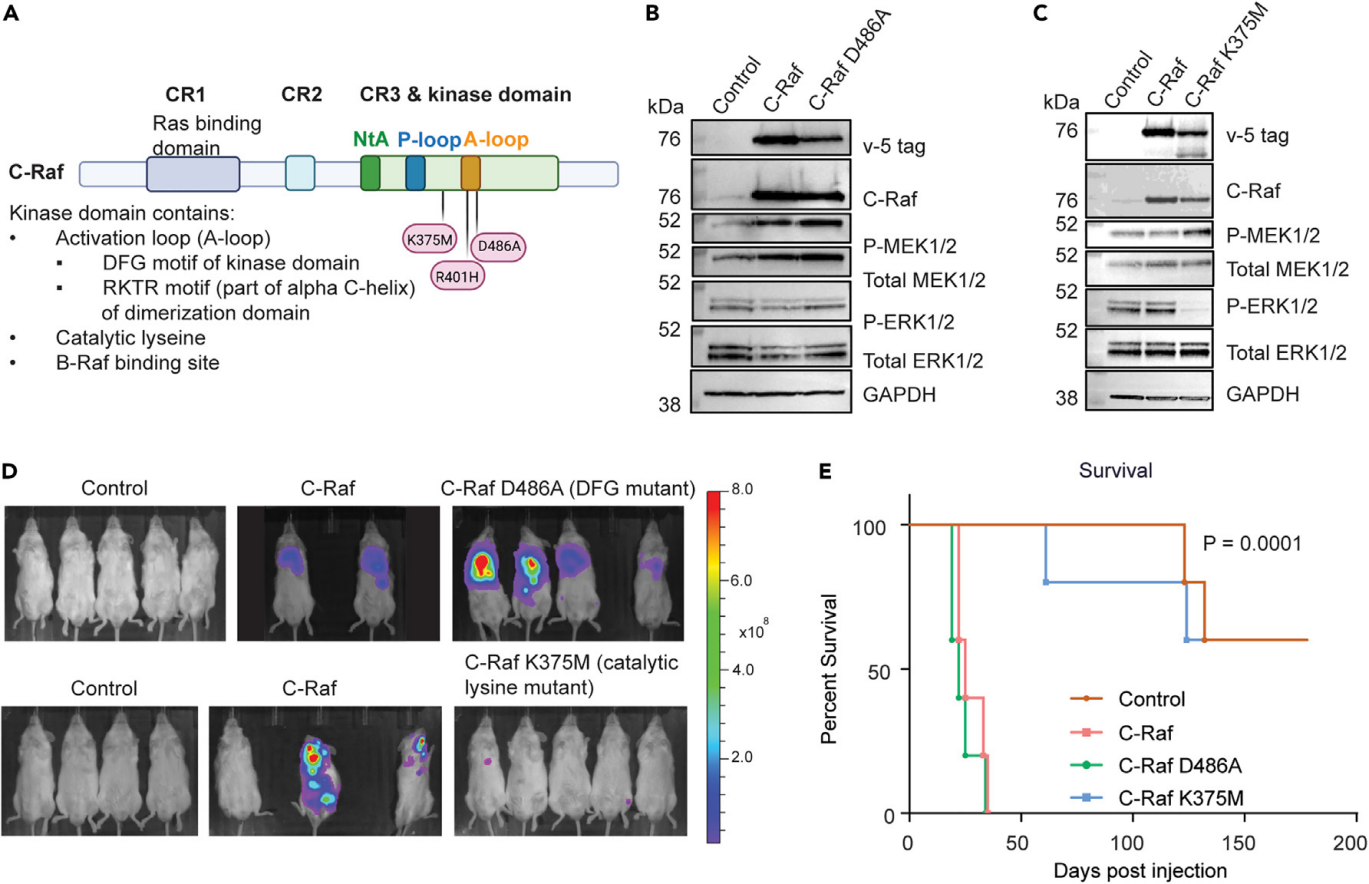
Mutation of the DFG motif and the ATP binding site in C-Raf’s kinase domain result in different metastatic phenotypes (A) Schematic of C-Raf structure indicating location of mutations relative to the rest of the protein structure. (B) Western blot depicting expression of C-Raf and C-Raf D486A mutant and downstream signaling. (C) Western blot depicting expression of C-Raf and C-Raf K375M and downstream signaling. (D) BLI imaging of both C-Raf kinase-dead mutants at 28 days post-intracardiac injection compared to controls. (E) Kaplan-Meier curve of mice harboring cells with vector control, C-Raf and C-Raf D486A and C-Raf K375M (n = 4/group Log rank (Mantel-Cox) test, p = 0.0001, all groups significantly different).

To determine whether C-Raf’s kinase activity is required for the metastatic phenotype, cell lines expressing C-Raf and two types of C-Raf kinase-dead mutants (D486A and K375M) were generated. C-Raf D486A mutant alters a key aspartate residue responsible for coordinating Mg2+ for ATP binding in the activation segment/Asp-Phe-Gly (DFG) motif of the kinase domain.^43^ C-Raf K375M mutant targets a catalytic lysine in the kinase domain responsible for mediating ATP catalysis^8^^,^^44^ (Figure 5A). Both mutations severely dampen C-Raf’s kinase activity.^37^^,^^43^^,^^45^^,^^46^ Previous work suggests that C-Raf D486A mutant may be unstable and degraded due to misfolding. Surprisingly, C-Raf D486A did not significantly affect phospho-MEK1/2 and phospho-ERK1/2 levels compared to WT C-Raf (Figure 5B), while expression of C-Raf K375M diminished phospho-ERK1/2, but not phospho-MEK1/2 levels (Figure 5C). This suggests that even with misfolding, D486A retains the ability to drive MAPK signaling through interaction with endogenous Raf molecules (Figure 5B).

**Figure 6:**
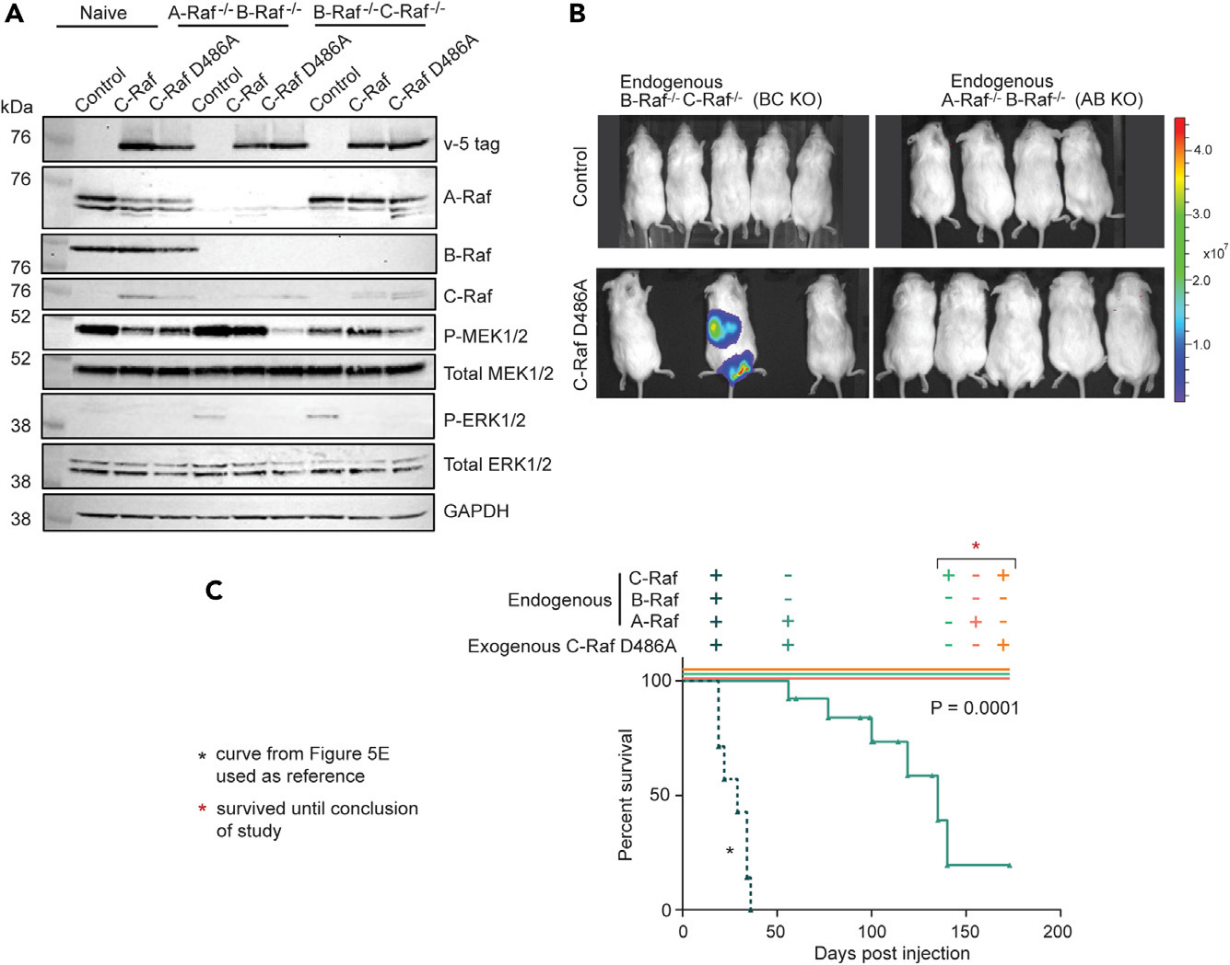
Overexpression of C-Raf DFG kinase-dead mutant requires endogenous Raf to drive metastasis (A) Western blot depicting C-Raf D386A mutant expressed in wild-type or combination AB KO or BC KO and downstream signaling. (B) BLI imaging at 70 days post-intracardiac injection of mice expressing vector control, C-Raf and C-Raf D486A in AB KO or BC KO backgrounds. (C) Kaplan-Meier curve of groups depicted in (B) (n = 8/group log rank (Mantel-Cox) test, p = 0.0001, all groups significantly different).

*In vivo* administration of C-Raf D486A resulted in robust metastasis, which significantly reduced survival to levels comparable to WT C-Raf (Figures 5D and 5E). In contrast, injection with C-Raf K375M-expressing cells resulted in significantly delayed metastatic latency compared to WT C-Raf, with some animals surviving until the end of the study (Figure 6E). These differing results for the two C-Raf kinase-deficient mutants indicate functional nuances in C-Raf’s kinase domain that may be independent of its kinase activity.

### Overexpression of C-Raf DFG kinase-dead mutant requires endogenous Raf to drive metastasis

Mutation of the catalytic lysine K375 is within the region of reported high affinity for B-Raf binding, while the DFG motif (D486A) is reported to have medium binding affinity.^44^ Since dimerization precedes kinase activation, the differences in metastatic phenotypes between these two mutants may be due to their differential effect on C-Raf’s ability to heterodimerize with B-Raf. Studies have shown that A- and B-Raf kinase mutants can interact with endogenous kinase-competent C-Raf monomers to drive aberrant signaling in cancer.^47^^,^^48^^,^^49^ We hypothesize that the C-Raf D486A mutation ablates kinase activity but does not detrimentally affect B-Raf binding. Endogenous B-Raf may dimerize with C-Raf D486A and compensate for its compromised kinase activity. To test this hypothesis, C-Raf D486A was expressed in BC KO cells and evaluated for its effect on metastatic output (Figure 6A). Mice harboring C-Raf D486A BC KO cells had significantly delayed metastatic growth and survived ∼110 days longer relative to C-Raf D486A (Figures 6B and 6C). While ablation of endogenous B-Raf and C-Raf significantly increased metastatic latency, the presence of endogenous A-Raf may cooperate with C-Raf D486A for weak metastatic output via the MAPK pathway. We expressed C-Raf D486A in AB KO cells to test the additional role of A-Raf in C-Raf D486A-driven metastasis. Unexpectedly, when the only available endogenous Raf monomer is C-Raf in the AB KO background, mice harboring C-Raf D486A had no metastatic lesions and survived to the conclusion of the study with no measurable tumors as assessed by BLI (Figures 6B and 6C). This experiment was repeated twice to ensure accuracy of results. The presence of endogenous A-Raf in BC KO cells can compensate for C-Raf D486A’s loss of kinase activity, presumably via dimerization. However, endogenous C-Raf, at low expression, may not be sufficient to rescue C-Raf D486A’s low kinase activity in AB KO cells, resulting in no metastasis (Figure 6C). These findings indicate that C-Raf’s intrinsic kinase activity may not be essential, provided that C-Raf can dimerize with kinase-competent endogenous Raf molecules to generate adequate MAPK signaling for promoting metastasis.

## Discussion

Cancer cells often exhibit heightened MAPK pathway activity due to activating mutations and Raf alterations.^3^^,^^50^^,^^51^ However, the role of WT Raf in metastasis remains unclear. This study explores the pivotal factors driving metastasis via elevated C-Raf signaling.

We discovered that non-mutated C-Raf’s metastatic potential hinges on elevated expression beyond endogenous levels. Various mechanisms can trigger this, irrespective of genomic amplification or activating mutations. Negative feedback mechanisms like ERK1/2-driven hyperphosphorylation of C-Raf, reversed by phosphatase PP2A and prolyl isomerase Pin1, can restore its signaling competence.^52^ PP2A activity shifts can result in accumulated active C-Raf. A similar scenario involving PTP1B elevation sensitizes renal cell carcinoma to SRC inhibition.^53^

Our findings highlight the pivotal role of C-Raf’s dimerization domain and its interactions in metastasis. The differing metastatic outcomes of C-Raf kinase domain mutants (K375M and D486A) likely arise from their impact on dimerization with B-Raf. Rushworth et al.'s work emphasizes distinct binding affinity regions in C-Raf’s kinase domain, with D486 in a medium-affinity and K375 in a high-affinity region for B-Raf binding (Rushworth et al.^44^). Since dimerization precedes kinase activation, the K375 mutation could impede dimerization with kinase-competent B-Raf, potentially limiting MAPK flux and cellular fitness, leading to reduced metastasis. Our findings suggest reduced downstream MAPK signaling in the K375 mutant compared to D486A, indicating an insufficient number of competent Raf signaling molecules for robust MAPK pathway activation. Therefore, the necessity of C-Raf’s kinase activity for metastasis might depend on other kinase-competent Raf paralogs, particularly B-Raf, to ensure sufficient MAPK pathway output.

Supporting this study, Venkatanarayan and colleagues have reported that the expression of C-Raf K375M mutant notably reduces B-Raf heterodimer formation. Despite this reduction, the mutant can still drive malignant phenotypes in K-Ras-driven cancer, possibly through the constitutive activity of mutant K-Ras.^13^ They also demonstrate that C-Raf K375M prefers dimerization with A-Raf, which has the weakest kinase activity among the three Raf kinases.^54^ This body of evidence suggests that C-Raf’s intrinsic kinase activity is dispensable for metastasis if it can compensate by dimerizing with other kinase-competent monomers.

Heterodimerization proves vital for C-Raf’s metastatic drive, evident from C-Raf D486A expression without endogenous B-Raf or A-Raf, significantly reducing or completely abolishing metastatic ability (Figure 4E). Consistent results were reported by Sanclemente et al. (2021), where a kinase-dead C-Raf rescued tumor regression upon C-Raf ablation in K-Ras mutant lung adenocarcinomas. Similarly, Venkatanarayan and colleagues highlighted the specific role of A-Raf:C-Raf dimer pairs in propagating mutant K-Ras signals *in vitro*. Collectively, these data suggest that non-mutated C-Raf contributes to the metastatic phenotype through C-Raf kinase-independent, dimerization-dependent signaling via the MAPK pathway.

C-Raf’s roles extend beyond the MAPK pathway, encompassing scaffold functions. Its MAPK-independent roles are substantiated by research that delves into its involvement in apoptosis, cell contractility, and migration, facilitated through interactions with proteins like Bad, Bcl-2, ASK1, MST2, and Rok-α.^55^^,^^56^^,^^57^^,^^58^^,^^59^ Further exploration of these interactions in the context of metastasis holds promise for future studies.

Next-generation allosteric type II Raf inhibitors, termed "paradox breakers," prevent pathway reactivation by targeting the DFG-out/αC-helix-in conformation with higher affinity for B-Raf mutants.^60^ Several of these inhibitors are in development, including those in early-stage clinical trials.^61^^,^^62^^,^^63^^,^^64^ Phase I trials of the pan-Raf inhibitor LY3009120 revealed toxicity and limited impact on Phospho-ERK inhibition, despite adequate drug levels in blood plasma.^64^ While pan-Raf inhibition remains crucial, our study and others emphasize the significance of Raf heterodimerization over homodimerization in mediating metastatic phenotypes.^13^^,^^40^^,^^44^^,^^65^^,^^66^ Future Raf inhibitor development may require heightened specificity to effectively target Raf-mediated metastatic signaling pathways.

### Limitations of the study

Our study defines the dimerization/interaction-dependent roles of Raf paralogs in C-Raf-driven metastasis. Specifically, our data support heterodimerization of C-Raf with either B-Raf or A-Raf as prominent MAPK signaling outputs to facilitate metastasis with C-Raf overexpression. Our findings do come with certain limitations. We were able to establish some correlative data regarding the necessity and requirement for downstream MAPK signaling. Further experimentation with conditional knockout or inhibitor studies of MEK1/2 can further delineate and demonstrate the dependency that C-Raf-driven metastasis has on downstream MAPK signaling. Additionally, to further support our claim and others’ that B-Raf is the most potent dimer pair, additional studies would benefit with interrogating the metastatic latency of C-Raf overexpression in BC knockout compared to C-Raf overexpression in AC knockout cells. Our survey of C-Raf interactors at the dimerization domain is focused on examining the Raf family kinases. Future studies would benefit from interrogation of how interactors like KSR1/2 MEK1/2, 14-3-3, CDC37, and HSP90 could affect metastasis. Our unbiased evaluation of the effects of ectopic C-Raf expression on the phosphoproteome and transcriptome surprisingly pointed strongly toward MAPK pathway upregulation. However, it would be interesting to explore these measurements using direct metastatic lesions, instead of cell lines or lines derived from metastatic lesions.

## STAR★Methods

### Key resources table

**Table:** 

REAGENT or RESOURCE	SOURCE	IDENTIFIER
**Antibodies**		

Mouse monoclonal Anti-V5	Thermo Fisher	Cat#: R960-25; RRID:AB_2556564
Rabbit monoclonal Anti-B-Raf (55C6)	Cell Signaling	Cat#: 9433; RRID:AB_2259354
Rabbit polyclonal Anti-A-Raf	Cell Signaling	Cat#: 4432; RRID:AB_2259354
Rabbit polyclonal Anti-RAF1	Sigma Aldrich	Cat#: HPA002640
Rabbit monoclonal Anti- Phospho-p44/42 MAPK (Erk1/2)	Cell Signaling	Cat#: 4370; RRID:AB_2315112
Rabbit monoclonal Anti- Phospho-MEK1/2	Cell Signaling	Cat#: 9154; RRID:AB_2138017
Rabbit monoclonal Anti- MEK1/2	Cell Signaling	Cat#: 8727; RRID:AB_10829473
Mouse monoclonal Anti- p44/42 MAPK (Erk1/2) (L34F12)	Cell Signaling	Cat#: 4696; RRID:AB_390780
Rat monoclonal Anti-GAPDH	Biolegend	Cat#: 607903; RRID:AB_2734504

**Critical commercial assays**		

CellTitre-Glo Luminescent Cell Viability Assay	Promega	Cat# G7573
QuikChange II Site-Directed Mutagenesis Kit	Agilent	200523

**Deposited data**		

RNA Sequencing hosted on GEO repository	This paper	Accession #: GSE235178
Raw and processed meta data from phosphoproteomic enrichment and LC/MS/MS hosted on Massive part of ProteomeXchange project server	This paper	Accession #: MSV000091647

**Experimental models: Cell lines**		

BPH-1	EMD Millipore	Cat#: SCC256
RWPE-1	ATCC	Cat#: CRL-11609D
RWPE-1 ILYW (Vector)	This study	N/A
RWPE-1 Ub-C-Raf	This study	N/A
BPH-1 ILYW-sGluc	This study	N/A
BPH-1 Ub-C-Raf-sGluc	This study	N/A
BPH-1 EFS-C-Raf-sGluc	This study	N/A
BPH-1 PGK-C-Raf-sGluc	This study	N/A
BPH-1 Ub-C-Raf D486A-sGluc	This study	N/A
BPH-1 Ub-C-Raf K375M-sGluc	This study	N/A
BPH-1 Ub-C-Raf R401H-sGluc	This study	N/A
BPH-1 B-Raf KO	This study	N/A
BPH-1 B-Raf, C-Raf KO ILYW	This study	N/A
BPH-1 B-Raf, A-Raf KO ILYW	This study	N/A
BPH-1 B-Raf, C-Raf KO C-Raf-sGluc	This study	N/A
BPH-1 B-Raf, A-Raf KO C-Raf -sGluc	This study	N/A
BPH-1 B-Raf, C-Raf KO C-Raf D486A-sGluc	This study	N/A
BPH-1 B-Raf, A-Raf KO C-Raf D486A-sGluc	This study	N/A
BPH-1 C-Raf Bone 1 Met	This study	N/A
BPH-1 C-Raf Bone 2 Met	This study	N/A
BPH-1 C-Raf Bone 3 Met	This study	N/A
BPH-1 C-Raf Bone 4 Met	This study	N/A
BPH-1 C-Raf Bone 5 Met	This study	N/A
BPH-1 C-Raf LN 1 Met	This study	N/A
BPH-1 C-Raf LN 2 Met	This study	N/A
BPH-1 C-Raf Spine 1 Met	This study	N/A
BPH-1 C-Raf Spine 2 Met	This study	N/A
BPH-1 C-Raf Thymus Met	This study	N/A

**Oligonucleotides**		

sgARAF – ACAATTTTGTGAGTGCAGGG	This study	N/A
sgBRAF – TTGAAGGCTTGTAACTGCTG	This study	N/A
sgRAF1 – GACCATGTGGACATTAGGTG	This study	N/A

**Recombinant DNA**		

FU-ILYW	This study	N/A
FU-ILYW-C-Raf	This study	N/A
FU-ILYW-C-Raf D486A	This study	N/A
FU-ILYW-C-Raf K375M	This study	N/A
FU-ILYW-C-Raf R401H	This study	N/A

**Software and algorithms**		

Prism 8	Graphpad	N/A
R version 3.4.0	The R Consortium	N/A
ImageJ	NIH	Version 1.51

### Resource availability

#### Lead contact

Requests for further information and reagents should be directed to and will be fulfilled by the lead contact, Owen Witte (owenwitte@mednet.ucla.edu).

#### Materials availability

Requests for plasmids, cell lines, and other reagents generated in the study should be directed to and will be fulfilled from the lead contact Owen Witte (owenwitte@mednet.ucla.edu), with a completed materials transfer agreement.

#### Data and code availability

• The RNA sequencing data has been deposited in the GEO repository and are publicly available as of the date of the publication. Accession numbers are listed in the key resources table. The raw data files for the mass spectrometry have been deposited to MassIVE database and are also publicly available as of the date of the publication. Accession numbers are listed in the key resources table.
• This paper does not report original code.
• Any additional information required to reanalyze the data reported in this work paper is available from the lead contact upon request.

### Experimental model and study participant details

#### Cell lines and cell culture

BPH-1 cells were propagated in RPMI supplemented with 10% (vol/vol) FBS (Gibco) and glutamine (1 mM). RWPE-1 cells were purchased from ATCC and cultured in keratinocyte serum-free medium (K-SFM) (Gibco) supplemented with 0.05 mg/mL bovine pituitary extract (Gibco), 5 ng/mL EGF (Gibco), penicillin (100 U/mL), and streptomycin (100 μg/mL). 293t cells used for lentiviral production were cultured in DMEM supplemented with 10% (vol/vol) FBS and glutamine (1 mM).

##### Animal studies and tumor models

All animal experiments were performed according to the protocol approved by the Division of Laboratory Medicine at the University of California, Los Angeles. NOD-scid gamma mice were purchased from Jackson Laboratories. For all experiments, male mice between 6 and 8 weeks of age were used. Mice were anesthetized at 2% (vol/vol) Isoflurane prior to intracardiac injection. A cell preparation was prepared at 0.250e6/injection of 100 uL in 1x PBS and injected into the left ventricle of the mouse heart. For subcutaneous cell injections, a cell preparation was prepared at 1e6/100 uL in 1x PBS and injected into the right flank of mice. Gaussia luciferase measurements were conducted weekly and were performed as described.^16^

### Method details

#### *In vitro* methods

##### Cell titer glo experiments

Proliferation experiments were performed following 5 days incubation in ultra-low attachment plates (Corning cat. CLS3471-24EA) and read using Promega Cell Viability assay (Promega cat. G7570).

##### Cloning

For cloning of the Ub-C-Raf overexpression vectors (C-Raf WT, C-Raf R401H and C-Raf D486A) C-Raf in C-Raf-ILYW (Falteirmeier et al. 2013) was mutated using site direct mutagenesis kit (Agilent 200523). Ub reporter was swapped out for EFS and PGK promoters via Gibson cloning. LentiCRISPR v2 CRISPR/cas9 system all-in-one dox inducible system was used to express cas9 and sgRNA targeting ARAF, BRAF and RAF1 genes. TLCV2 (Addgene #87360) was generated by insertion of ARAF, BRAF and RAF1 guide RNA (sgARAF – ACAATTTTGTGAGTGCAGGG, sgBRAF – TTGAAGGCTTGTAACTGCTG, and sgRAF1 – GACCATGTGGACATTAGGTG) into TLCV2 vector. All cloning was sequence verified.

##### Virus production

Third-generation lentiviruses were prepared by calcium phosphate precipitation transfection of 293t cells with plasmids expressing kinases with firefly luciferase reporter gene (FU-ILYW), Cas9 and guide RNAs (TLCV2), and gaussian luciferase plasmid (CMV-Gluc-IRES-GFP) (Targeting systems cat. GL-001). The lentiviruses were prepared as described.^67^ Viruses were tittered using serial transduction protocol of naïve 293t and assessed via flow cytometry using YFP and GFP.

##### Clonal knock out using CRISPR/Cas9

BPH-1 cells were infected with B-Raf-TLCV2 virus at an MOI of 10 for 48 hours. Infected cells were then treated with puromycin for selection of plasmid positive cells at 1 ug/mL for 72 hours. After puromycin selection, cells were then treated with 1 ug/mL of doxycycline for 72 hours to induce Cas9 expression and cutting. Cells were then expanded and then plated into 96 wells at once cell/well. Clones were then individually grown out, and screened via western blot for changes in protein levels. Clones that exhibited diminished or non-existent specific protein bands were additionally DNA sequenced at the site of the CRISPR edit to determine heterozygous or homozygous frame shift.

##### Western blot

Whole-cell lysates were prepared in Urea lysis buffer (8M Urea, 4% CHAPS, cOmplete™ Protease Inhibitor Cocktail from Roche with phosphatase inhibitor). Equal amounts of protein were separated by 4–12% (mass/vol) Bolt™ 4 to 12%, Bis-Tris SDS/PAGE (Thermo Fisher), followed by immunoblotting analysis with the indicated antibodies. The following antibodies were used to detect the corresponding proteins V5 (Invitrogen R960-25; 1:2,500); BRAF (Cell Signaling 55C6; 1:1,000); ARAF (Cell Signaling 4432S; 1:1000); C-Raf (Sigma HPA002640; 1:1000); Phospho-p44/42 MAPK (Erk1/2) (Cell Signaling 4370S; 1:1000); Phospho-MEK1/2 (Cell signaling 9154S; 1:1000); MEK1/2 (Cell Signaling 8727S; 1:1000); p44/42 MAPK (Erk1/2) (L34F12) (Cell Signaling 4696S; 1:1000); GAPDH (Biolegend 607903; 1:2,500).

##### Phospho-proteomics

Cells were grown at 70% confluency (10e6) harvested and washed 2x with ice cold PBS. Cells were subsequently scaped off using a cell scraper, pelleted and washed 2x with ice cold PBS. Cell pellets were lysed in Urea lysis buffer (8M Urea, 100mM Tris pH8.5, AEBSF, phosphatase inhibitor, benzonase and 1mM DTT) and incubated at RT for 0.5 hour. Lysates were cleared by centrifuging at 12000 g for 15 minutes. Supernatants from samples were transferred to a set of new tubes and concentrations were determined by absorbance at 280nm. 150ug of protein was taken from each sample and proceeded to reduction (5mM TCEP) and alkylation (10mM iodoacetamide). Reduced and alkylated protein samples were cleaned up by SP3 method,^68^ then 0.2ug of Lys-C and 2ug of Trypsin proteases were added to each sample and digestion were conducted for overnight. Digested peptide samples were labeled by 0.3ug of TMT isobaric tagging reagent for 1 hour and quenched by adding hydroxylamine to 0.5%. Equal amount of labeled peptide from each sample was pooled. The pooled mixture was used to perform phospho-peptide enrichment using Thermo High-Select Fe-NTA phospho-peptide enrichment kit (A32992). Finally, enriched TMT-labeled phospho-peptides were fractionated by CIF method^23^ to 6 fractions.

##### LC-MS acquisition

A 75 μm x 25 cm homemade C18 column was connected to a nano-flow Dionex Ultimate 3000 UHPLC system. The 70-minute gradient of increasing acetonitrile (ACN) was delivered at a 200nl/min flow rate as follows: 1% ACN phase from minutes 0 – 6, 6 - 25% ACN from minutes 6 – 55, 25 - 32% ACN from minutes 55 - 63.5, 32 - 80% ACN from minutes 63.5 – 67, and then 1% ACN from minutes 68 - 70. An Orbitrap Fusion Lumos Tri-brid mass spectrometer was used for data acquisition in TMT-SPS-MS3^69^ mode. Full MS scans were acquired at 120K resolution with the AGC target set to standard and a maximum injection time set to 50 ms. MS/MS scans were collected in linear ion trap in Turbo mode after isolating precursors with an isolation window of 0.7 m/z and CID-based fragmentation using 35% collision energy. Synchronized precursor selection was performed and 10 precursors were fragmented with 75% energy of HCD and sent for MS3 scans in Orbitrap with 50K resolution. were collected. For data dependent acquisition, a 3-second cycle time was used to acquire MS/MS and MS3 spectra corresponding to peptide targets from the preceding full MS scan. Dynamic exclusion was set to 30 seconds.

##### Mass spectrometry data analysis

MS/MS database search was performed using MaxQuant (1.6.10.43) against the human reference proteome from EMBL (UP000005640_9606 HUMAN Homo sapiens, 20874 entries). The search included carbamidomethylation on cysteine, TMT isobaric tag on lysine and peptide N-terminus as a fixed modification. Serine, threonine and tyrosine phosphorylation, methionine oxidation and N-terminal acetylation were set as variable modifications. The digestion mode was set to trypsin and allowed a maximum of 2 missed cleavages. The precursor mass tolerances were to 20 and 4.5 ppm for the first and second searches, respectively. Datasets were filtered at 1% FDR at the PSM level. Peptide quantitation was performed using MaxQuant’s multiplexing TMT 10plex MS3 mode. Site level t-test was performed using summarized phosphosites quantitation output from MaxQuant. Differentially (p-value<=0.05) quantified peptides from each pair of comparison were sent to PhosFate^25^ to infer kinase activity change.

#### Transcriptional profiling of C-Raf cell lines

##### RNA-sequencing

Transcriptomic profiling was performed using the TOIL pipeline. The transcriptomic dataset was filtered for coding genes with low additional filtering of variance and low abundance. We used ComBat-seq^70^ to adjust for batch effects attributed to tumors coming from two different cell lines/models. Expected counts were log2 transformed. After processing, the final transcriptomic dataset consisted of 10,958 genes.

##### Differential RNA abundance

DESeq2^71^ was used to perform differential RNA abundance analysis. Comparisons were performed between parental C-Raf vs vector control, which identified 667 differentially abundant genes, and C-Raf metastasis derived line vs parental C-Raf, which identified 634 differentially abundant genes. Statistical significance was determined using FDR-adjusted p-value < 0.05. Using these sets differentially abundant genes, we identified 46 genes that were perturbed in a stepwise fashion. That is, these genes were altered in the same direction for both comparisons.

##### Hallmarks signature scoring

To evaluate well-characterized biological processes, we scored each sample for the 50 Hallmarks gene sets.^72^ The transcriptomic dataset was first normalized by z-scoring across samples and across genes, then scored by taking each gene in the Hallmarks gene sets and median dichotomizing the samples. Samples with RNA abundance levels greater than the median were assigned a score of +1 for the gene, and samples with RNA abundance levels lesser than the median were assigned a score of -1 for the gene. This was repeated for every gene in the gene set, and the scores were summed for each sample to give the sample’s signature score. The volcano plot, heatmap and boxplots were generated using the BoutrosLab.plotting.general R package.^73^

#### *In vivo* methods

##### Bioluminescence imaging

Bioluminescence Imaging (BLI) was conducted using an IVIS Lumina II (PerkinElmer). D-luciferin (150 mg/kg) was injected intraperitoneally. After 15 min, anesthetized mice (using 2% (vol/vol) isoflurane) were imaged. BLI analysis was performed using Living Image software, version 4.0 (PerkinElmer).

### Quantification and statistical analysis

Western blot quantification was assessed using densitometry through ImaeJ software. Significance was determined at p < 0.05 and denoted as ∗, ∗∗, ∗∗∗, and ∗∗∗∗. '+' signifies 0.05 > p > 0.1. Figure legends provide details about the applied statistical tests. Two-sided t-tests were used for normally distributed data in two-group comparisons, while two-sided Wilcoxon rank sum tests were employed for non-normal data or small sample sizes with strong outliers. Data are typically presented with error bars depicting ±1 standard deviation and SEM.
