# Supplementary material for: Wild-type C-Raf gene dosage and dimerization drive prostate cancer metastasis
Source: iScience. 2023 Nov 17;26(12):108480. doi: 10.1016/j.isci.2023.108480 (PMC10711388; doi:10.1016/j.isci.2023.108480)
Supplement: Document S1. Figures S1–S4 [file mmc1.pdf]

iScience, Volume *26*

Supplemental information

Wild-type C-Raf gene dosage

and dimerization drive prostate cancer metastasis

Lisa Ta, Brandon L. Tsai, Weixian Deng, Jihui Sha, Grigor Varuzhanyan, Wendy Tran, James A. Wohlschlegel, Janai R. Carr-Ascher, and Owen N. Witte

SUPPLEMENTAL FIGURES

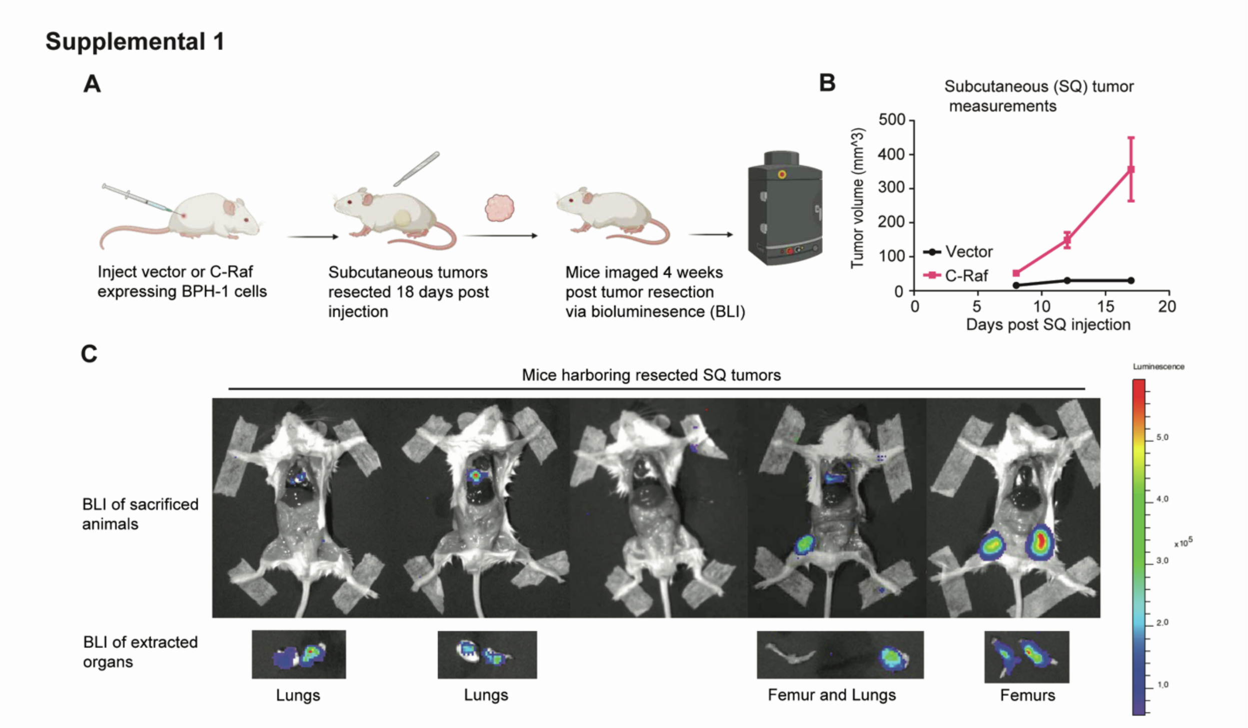

Supplemental Figure 1: C-Raf drives spontaneous metastasis to the lung and bone

A) Experimental schematic of experimental spontaneous metastasis. Mice were injected subcutaneously (SQ) with 1e6 cells vector or C-Raf expressing BPH-1 cells and monitored over 2 weeks for SQ tumor growth (n=5/group) **B)** Caper measurements over a 2 weeks of C-Raf or vector expressing BPH-1 cells **C)** BLI image of mice administered 150 mg Luciferin/kg body weight and subsequently sacrificed and imaged for metastatic lesions

Supplemental 2

A

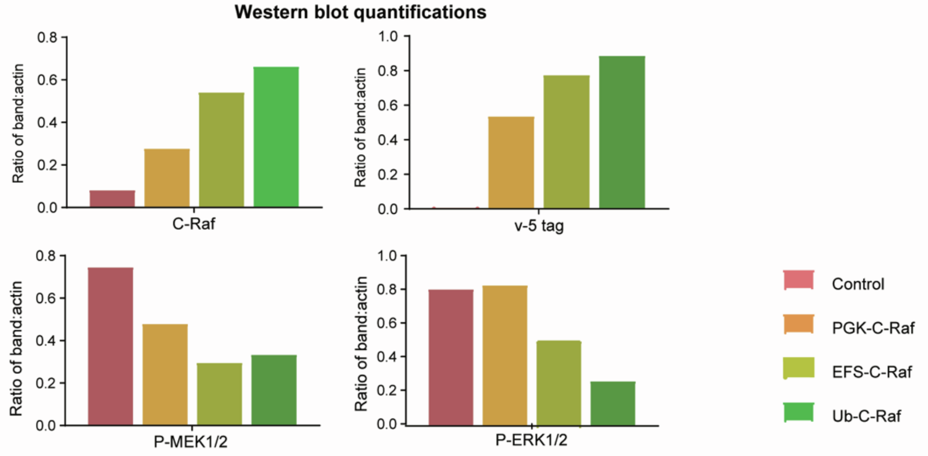

Supplemental Figure 2: C-Raf protein dosage western quantification

A) Densitometric quantification of C-Raf protein dosage protein expression and downstream effectors.

Supplemental 3

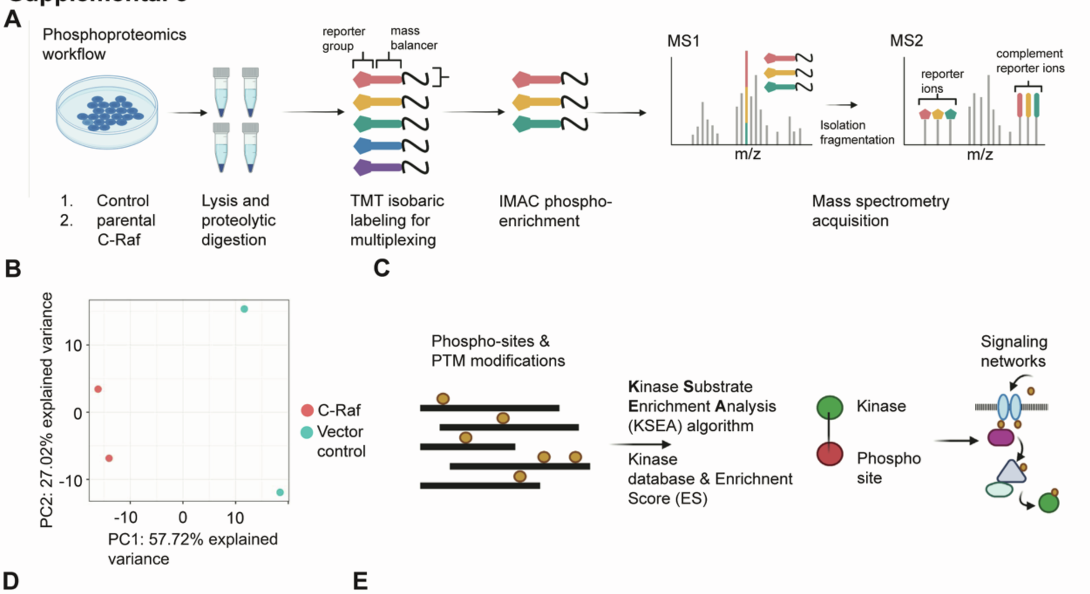

D

Upregulated kinases in C-Raf vs Vector control

Upregulated kinase activities	Identified Substrates in dataset
CDK2	28
VRK1	3
VRK2	3
HIPK2	1
PRKG1	3
CAMK2A	1
CAMK2B	1
CDK1	21
CAMK4	1
RAF1	3
SGK1	1
LATS1	1
MAPKAPK2	3
PLK1	1
PRKDC	1
AKT2	1

0
10
20
30
40
60

$p < 0.05$

E

pvalue	Pathway	quantSites	proteins
0.001	RAF1-RAS complex, EGF induced	5	4
0.002	P-TEFb-BRD4-TRAP220 complex	6	4
0.002	DDB complex	2	2
0.003	Ubiquitin E3 ligase (DDB1, DDB2, CUL4A, CUL4B, RBX1)	2	5
0.007	PTIP-HMT complex	1	10
0.017	Chromatin remodeling complex (TACC2, TACC3, PCAF)	1	3
0.017	Menin-associated histone methyltransferase complex	1	7
0.018	ITAGV-ITGB3-F11R complex	2	3
0.026	NFAT-JUN-FOS DNA-protein complex	2	3
0.03	ITGA2B-ITGB3-F11R complex	2	3
0.038	GR-hnRNP U complex	1	2

Supplemental Figure 3: C-Raf overexpression increases MAPK pathway flux and is associated with metastasis.

A) Phospho-proteomic workflow using tandem mass tag (TMT) isobaric labeling and mass spectrometry for multiplexing. Cells are lysed using proteolytic digestion and subsequently labeled with TMT isobaric labeling to allow for sample multiplexing. Samples simultaneously underwent Immobilized metal affinity chromatography (IMAC) phospho-enrichment and subsequent MS/MS analysis to determine the phosphoproteome relative to each other. **B)** Principal component analysis of phospho-proteomic samples captured a total of 84.74% of

variance in PC1 and PC2. **C)** Schematic of inferred kinase activity (IKA) analysis. **D)** List of top 15 upregulated kinases in C-Raf samples compared to vector control. **E)** Top 10 pathways upregulated via pathway flux analysis derived from inferred kinase hits with p value < 0.05.

Supplmental 4

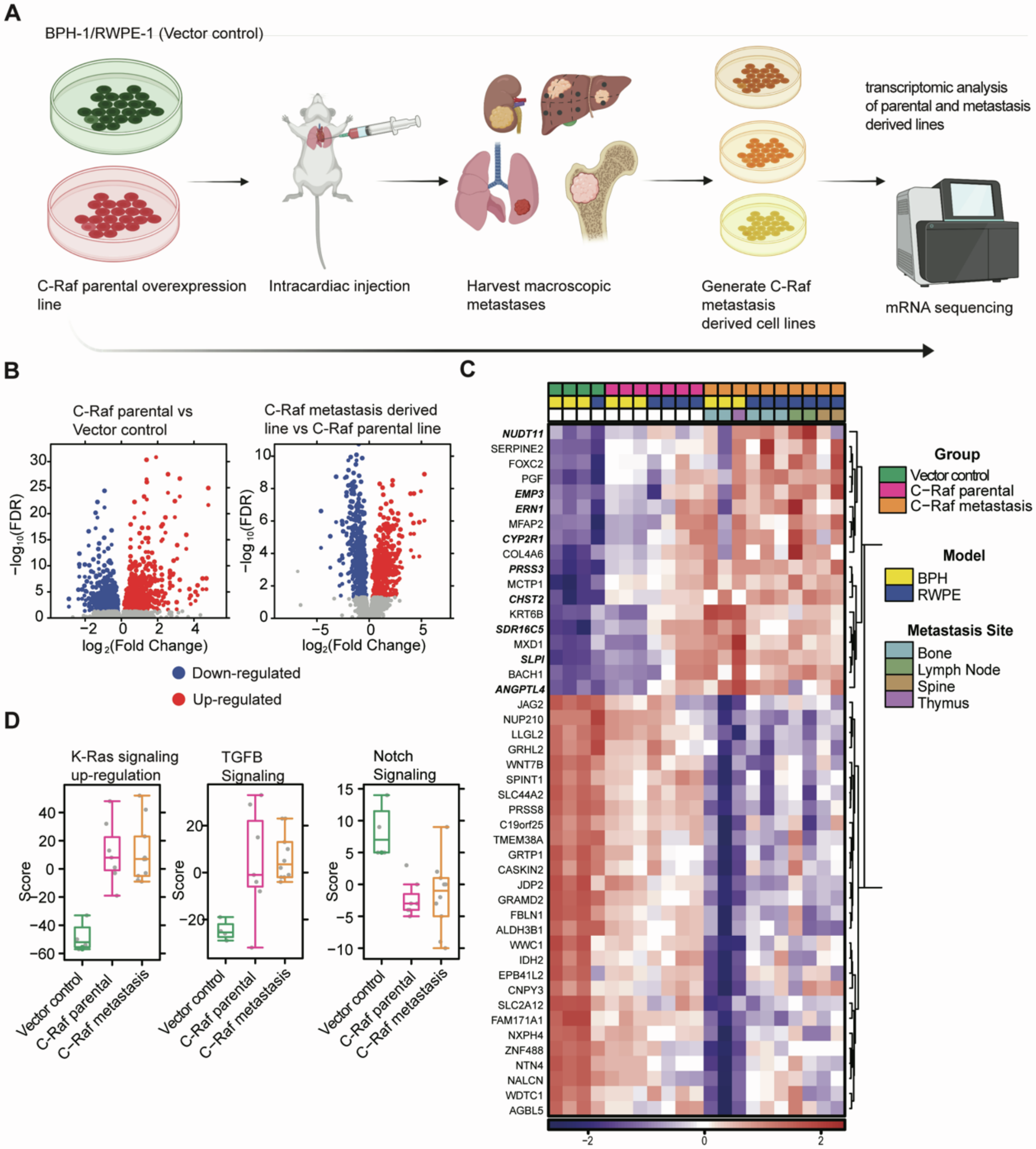

Supplemental Figure 4: Transcriptional targets of the MAPK pathway are upregulated in metastatic C-Raf driven tumors

A) Schematic of C-Raf metastasis derived cell line generation and subsequent mRNA sequencing analysis compared to parental cell lines. Cell lines were generated from bone, lymph, thymus, spine, and liver metastases. **B)** Volcano plots of genes altered upon C-Raf addition compared to vector control and C-Raf metastasis cell lines compared to C-Raf parental line. Statistical significance was determined using FDR <0.05. **C)** 46 differentially expressed genes that were perturbed in a stepwise fashion across parental and metastasis derived lines in BPH-1 and RWPE-1 (increasing and decreasing). Bold italicized genes indicate genes that are part of the Ras/Raf signaling pathway that increased from vector to C-Raf parental to C-Raf metastasis derived cell line. **D)** Significantly altered cancer hallmark pathway analysis using GSEA across sample groups (unpaired t - test, $p = 0.005$).
